# Total Pancreatectomy for Multicentric Cystic Neuroendocrine Tumor of the Pancreas: A Case Report

**DOI:** 10.3390/diagnostics12041003

**Published:** 2022-04-15

**Authors:** Milica Mitrovic-Jovanovic, Nikica Grubor, Stefan Milosevic, Aleksandra Jankovic, Katarina Stosic, Slavenko Ostojic, Aleksandar Ninic, Marjan Micev, Jelena Djokic Kovac

**Affiliations:** 1 Center for Radiology and Magnetic Resonance Imaging, University Clinical Centre of Serbia, Pasterova No. 2, 11000 Belgrade, Serbia; milosevic.stefan92@gmail.com (S.M.); jankovicm.alex@gmail.com (A.J.); katestosic@gmail.com (K.S.); jelenadjokickovac@gmail.com (J.D.K.); 2 Department for HBP Surgery, Clinic for Digestive Surgery, University Clinical Centre of Serbia, Koste Todorovica Street No. 6, 11000 Belgrade, Serbia; gruborn13@gmail.com (N.G.); slavenko.ostojic@gmail.com (S.O.); ninic.aleksandar89@gmail.com (A.N.); 3 Department for Surgery with Anesthesiology, Faculty of Medicine, University of Belgrade, Dr Subotica No. 8, 11000 Belgrade, Serbia; 4 Department for Pathology, Clinic for Digestive Surgery, University Clinical Centre of Serbia, Koste Todorovica Street No. 6, 11000 Belgrade, Serbia; micevm@gmail.com; 5 Department for Radiology, Faculty of Medicine, University of Belgrade, Dr Subotica No. 8, 11000 Belgrade, Serbia

**Keywords:** pancreatic neuroendocrine tumor (PNET), cystic PNET, MDCT, total pancreatectomy

## Abstract

Pancreatic neuroendocrine tumors (PNETs) are uncommon pancreatic neoplasms with malignant potential, heterogeneous clinical behavior, as well as imaging appearance. These tumors represent less than 3% of all pancreatic neoplasms with typical CT presentation as solid, well-circumscribed, hypervascular lesions. Cystic PNET is a rare pancreatic tumor which is nowadays more often detected due to the widespread use of high-resolution cross-sectional imaging. They are mainly solitary lesions most commonly localized in the body and the tail of the pancreas. Due to cystic presentation these lesions often present a diagnostic challenge to both experienced radiologists and pathologists. Herein, we present a rare case of synchronous, multiple cystic and solid pancreatic neuroendocrine tumors, which due to their extensiveness required total dudenopancreatectomy with splenectomy. Histopathological findings confirmed microscopic and macroscopic cystic components as well as typical solid variants of neuroendocrine tumors along the entire pancreas.

## 1. Introduction

Pancreatic neuroendocrine tumors (PNET) are relatively uncommon lesions, presenting fewer than 3% of all pancreatic neoplasms [1]. They are predominantly solid tumors with malignant potential, heterogeneous clinical behavior, as well as imaging appearance. Some cases are associated with familial syndromes such as multiple endocrine neoplasia type 1 (MEN1), von Hippel-Lindau syndrome (VHL), and neurofibromatosis type 1 [2]. Cystic forms of PNET are rarely seen in clinical practice with more favorable prognosis than solid PNET [3]. Recent studies showed that cystic PNET tend to have lower tumor grade, less aggressive behavior and better long-term prognosis [3]. According to the American Joint Committee on Cancer (AJCC) and European Neuroendocrine Tumor Society (ENETS) guidelines, cystic PNET often present lower pathologic stage and decreased Ki-67 proliferation index compared with solid counterpart. Rarely cystic PNET may be seen as multiple lesions with diffuse parenchymal distributions as in our case [4,5].

According to the ENETS consensus guidelines, the grading of proliferative rate of the NET cells should be based on the mitotic rate and Ki-67 labeling index. The newest World Health Organization (WHO) classification of NET includes grading and staging, and gives a basis for predicting prognosis of the disease [6].

However, accurate preoperative diagnosis of cystic PNETs still remains a challenge because they can be easily confused with other cystic pancreatic neoplasms. Herein, we present a very rare case of synchronous multicentric pancreatic cystic and solid forms of neuroendocrine tumors.

## 2. Case Report

**Figure 1:**
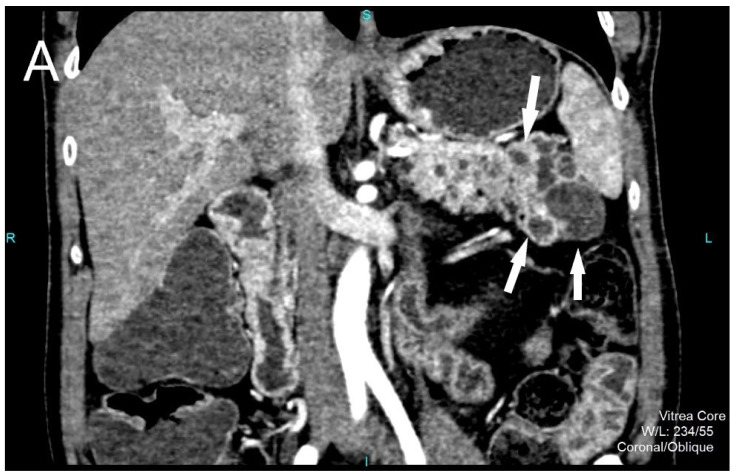

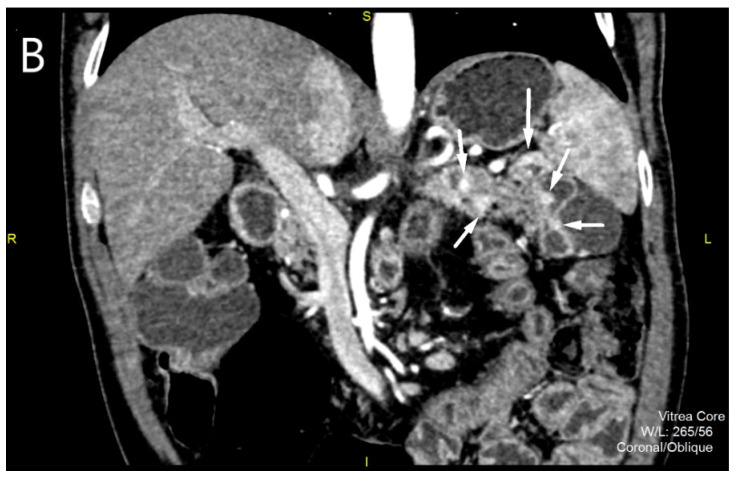
Contrast-enhanced computed tomography, coronal reconstruction, arterial phase of the exam reveals numerous confluent cystic lesions (white arrows) in the region of pancreatic tail and body (**A**) and also multiple small scattered solid hypervascular tumors (white arrows) (**B**).

A 49-year-old male patient was admitted to our hospital complaining about epigastric pain which lasted a few months. Upper gastro endoscopy revealed no abnormalities. Initial ultrasound examination showed multiple anechoic lesions measuring up to 20 mm, in the region of pancreatic tale and body. Laboratory tests, amylase, lipase, leukocyte formula and inflammatory markers were all within normal limits. Chromogranin A level was elevated up to 306.9 ng/mL while CA 19-9 was within normal limits. The patient underwent further abdominal computed tomography (CT) examination which showed multiple cystic lesions involving pancreatic tail and body measuring up to 20 mm. On arterial phase, a clear enhancement of irregular cystic wall was detected, while in the parenchyma between cystic lesions, multiple small solid hypervascular tumors were found (Figure 1A,B). The surrounding peripancreatic adipose tissue was normal. There were no signs of secondary dissemination.

**Figure 2:**
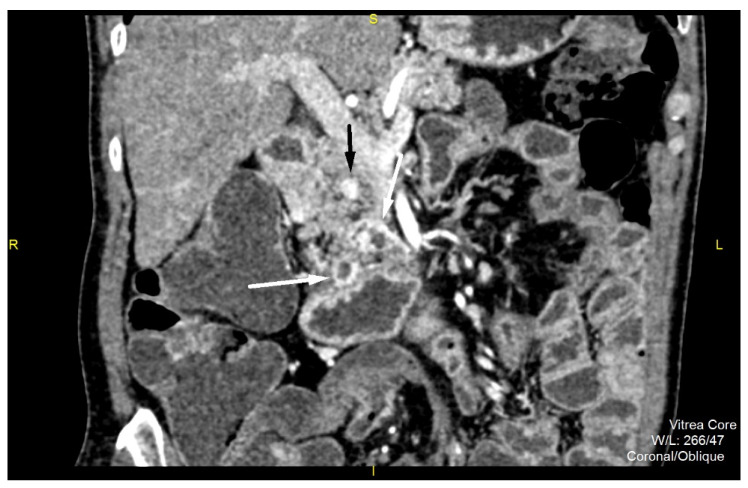
Multidetector computed tomography, arterial phase of the exam shows cystic lesion with tick, hyperdense wall inside the pancreatic head (white arrow) and nodular, hypervascular lesion (black arrow) in cranial aspect.

**Figure 3:**
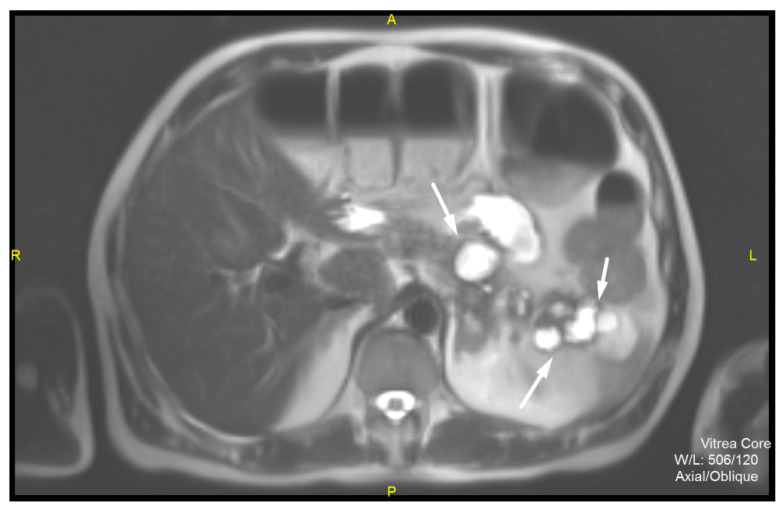
MR examination. On T2-weighted image multiple cystic lesions (white arrows) in the pancreatic body and the tail with thickened hypointense wall are seen. There is no dilatation of the main pancreatic duct, nor clearly visible connection of the cystic lesions with pancreatic ductal system.

Similar CT findings were also observed in the uncinate process (Figure 2). The MRI examination revealed multiple cystic lesions, especially in the region of the head and the tail of the pancreas without communication with the main pancreatic duct (Figure 3). There was no dilatation of the main pancreatic duct. Multiple small solid hypervascular tumors were also noted, similarly to CT. However, only in correlation with CT where intense enhancement of the wall of the cystic lesions was noted, and increased levels of chromogranin, the preoperative diagnosis of synchronous multiple cystic NET in combination with solid NETs was suggested.

**Figure 4:**
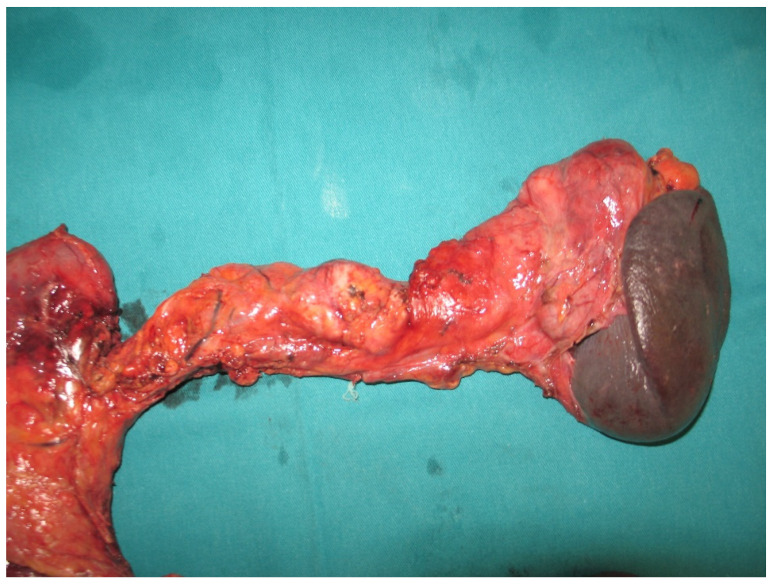
Macroscopic specimen after duodenopancreatectomy with splenectomy reveals multicentric neuroendocrine tumors distributed throughout whole pancreas.

Since the patient had multiple cystic and solid lesions distributed diffusely throughout pancreatic parenchyma, total pancreatectomy was performed. As the tail of the pancreas was voluminous, and in close relationship with the hilus of the spleen, the intraoperative decision was to perform a splenectomy as well (Figure 4).

**Figure 5:**
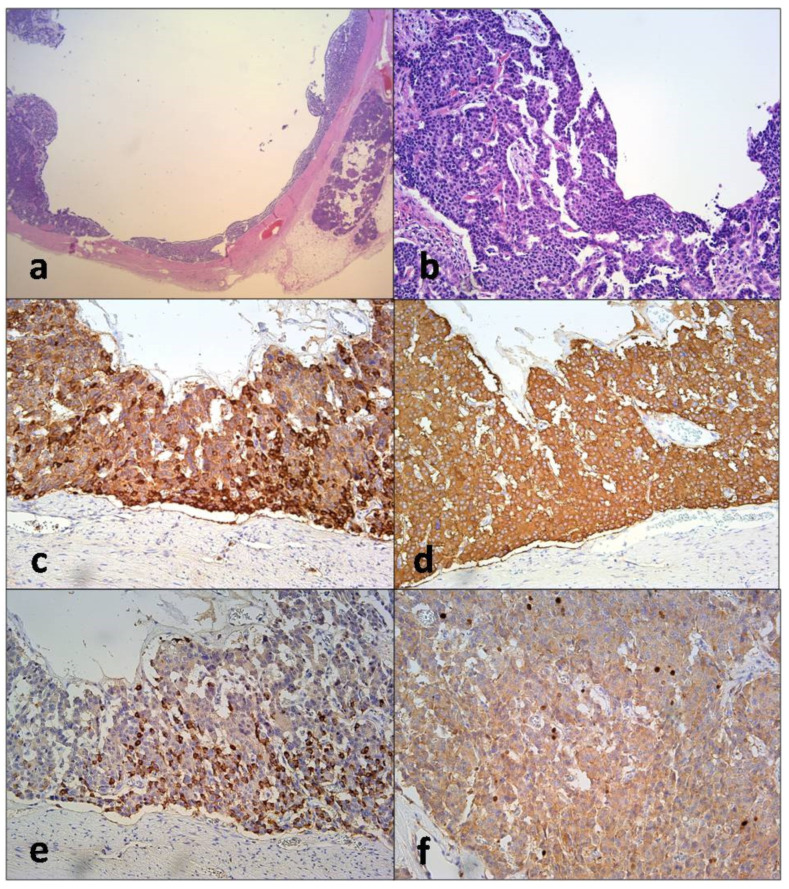
Microscopic cystic presentation of neuroendocrine tumor on low power magnification (**a**) with characteristic histological aspect of „carcinoid“ morphology on the inner surface (**b**). Immunohistochemistry revealed strong expression of chromogranin A (**c**) and synaptophysin (**d**) as well as focal immunoexpression of glucagon in small proportion of tumoral cells (**e**). The proliferative activity expressed by Ki-67 protein index was 6.8% (**f**).

Histopathological and immunohistochemical findings confirmed the diagnosis of multiple cystic and solid well-differentiated neuroendocrine tumors grade 2 (G2) (Figure 5). The patient successfully recovered and was dismissed from hospital twelve days after operation. Regular follow-up which included CT examination and chromogranin levels measurement every three months did not show disease reccurence one year after the operation. Due to the association of multicentric neuroendocrine tumors with familial syndromes, the patient underwent gene analyses.

Mutational analysis was performed on genomic DNA, extracted from peripheral blood leukocytes using Pure Link Genomic DNA Mini Kit (Termo Fisher Scientific, Waltham, MA, USA), according to the manufacturer’s instructions. The entire coding region plus flanking splice sites of MEN1 (exons 2–10) were analyzed by PCR sequencing using specific primers. Direct DNA sequencing using the Big Dye Terminator v3.1 Ready Reaction Cycle Sequencing Kit (Applied Biosystems, Waltham, MA, USA) was performed on automated ABI PRISM 3130 Genetic Analyzer (Applied Biosystems, Waltham, MA, USA) and analyzed with ABI DNA Sequencing Analysis Software v5.2. As the patient was negative for point mutations he was screened for larger deletions in MEN1 gene, using the SALSA MLPA P017-C1 MEN1 kit (MRC-Holland, Amsterdam, The Netherlands).

Since genetic analyzes did not show any abnormalities the patient was considered to have sporadic, nonfamilial multicentric PNETs.

## 3. Discussion

Pancreatic neuroendocrine tumors are rare tumors originating from pancreatic islet-cells [7]. Incidence of pancreatic NET is estimated to be less than 1 case per 100,000 [8]. PNET are mostly solid tumors, however in some cases, they can show cystic degeneration presenting as cystic lesion. Cystic PNETs comprise approximately 7–17% of all PNET, they are nonfunctional and mostly incidentally discovered [9].

PNET are mainly sporadic, nonfamilial tumors and only 10–15% can be associated with some familial cancer syndrome [10]. These hereditary PNETs can be seen in multiple endocrine neoplasia type 1 (MEN-1), von Hippel-Lindau syndrome, neurofibromatosis type 1, tuberous sclerosis, and nonpolyposis colon cancer. Although a relatively small percentage of NETs are inherited, a study conducted by Hasan et al. found that 54% of patients with NET had a first-degree relative with some type of malignancy [10,11]. Intrafamilial phenotypic variable expressivity in these syndromes indicate that the disease is affected also by environmental and personal risk factors, that have yet to be discovered. The era of somatic molecular analysis will bring identification of novel biomarkers for prognosis and new treatment targets for PNETs [12].

PNET clinical symptomatology is dependent on tumor hormonal secretion. All NET are divided on functional and non-functional based on their endocrine function, with only one quarter being hormonally active [13]. Non-functional PNETs are usually characterized by nonspecific symptoms, such as abdominal pain, nausea, weight loss or palpable abdominal mass, which frequently leads to their late diagnosis [13]. Biochemical biomarkers used in the diagnosis, prognosis assessment and therapeutic monitoring of PNET are divided into two groups of non-specific and specific markers. While specific markers indicate the presence of functional NET, non-specific markers such as chromogranin A, neuron-specific enolase, pancreatic-polipeptide, human chorionic gonadotropin and alpha-fetoprotein are useful in the diagnosis of non-functional NET [14].

Imaging has an important role in the diagnosis of PNET, especially in the incidental discovery of non-functional NETs. Different imaging modalities can be used, such as ultrasound, CT, MRI, somatostatin receptor scintigraphy and positron emission tomography (PET) [13]. Endoscopic ultrasound (EUS) combines both endoscopic and ultrasound examination into a single modality with high sensitivity and specificity in identifying PNETs. EUS offers the additional benefit in terms of obtaining biopsies and cyst fluid examination for additional pathological findings. Due to the high rate of diagnostic accuracy and low rate of complications, EUS has become an integral part of the preoperative assessment of pancreatic cysts [15]. Ultrasonographically, PNETs are usually seen as homogenous hypoechoic lesions, although larger tumors can be heterogeneous [13]. On CT, PNETs are commonly seen as focal well-circumscribed hyperdense lesions in arterial phase, due to their rich vascularization [16]. In portal-venous phase, they typically maintain postcontrast enhancement. Pancreatic duct dilation is not an obligatory finding in patients with pancreatic NET. MRI characteristics of solid PNETs include hipointensity on T1-weighted images, mild hyperintensity on T2-weighted images compared to pancreatic parenchyma, and intensive enhancement after contrast administration [17]. Restricted diffusion is frequently present and tends to correlate with degree of tumor differentiation [18]. Nuclear medicine imaging modalities such as PET and SPECT can be useful in detecting PNET metastasis as well as for the precise localization of primary tumor [19]. In our case, multiple small hypervascular pancreatic lesion were detected on both CT and MRI, which indicated the presence of multicentric NET. In contrast to solid PNET, the diagnosis of cystic PNETs presents a diagnostic challenge since these lesions may simulate other cystic pancreatic tumors [20]. Cystic PNETs usually arise in pancreatic body and tail, however in our patient a rare diffuse parenchymal distribution was present. Characteristic imaging feature of cystic PNET is their peripherally enhancing rim seen in arterial phase on CT and MRI [16,20]. Most cystic PNET are purely cystic, but in some cases, they can show mixed solid-cystic structure [21]. Calcification can be rarely seen, and is present in 13% of cystic PNETs [22]. In the study by Kaosombatwattana et al. a rare case of solitary cystic PNET was presented with an unusual egg-shell like wall calcification and dense internal content corresponding to intralesional necrosis and hemorrhage [23]. In our case, calcifications were not detected, and all cystic tumors had clear internal liquid content measuring up to 10 Hounsfield units.

Differential diagnosis of cystic PNET is complex and include intraductal papillary mucinous neoplasms (IPMNs), sequelae of an earlier inflammatory process, serous cystadenomas (SCAs), and mucinous cystic neoplasms (MCNs). IPMN can be excluded with magnetic resonance cholangiopancreatography (MRCP), since cystic PNET usually does not have communication with main pancreatic duct [22]. Also, IPMN does not show characteristic rim enhancement similarly to postinflammatory collections, SCA and MCN [23]. All cystic PNETs in our case showed intense wall enhancement in arterial phase. Therefore, it can be suggested that peripheral rim enhancement could be used as the most important characteristic for the differentiation of these rare tumors from other pancreatic cystic lesions. This also indicates the importance of accurate timing of early arterial phase on CT and MRI in the diagnosis of cystic PNET [7]. Moreover, the morphology of cystic tumors must be considered. While branch duct IPMN lesions show “bunch of grapes” appearance, cystic NETS tend to be unilocular and round. On the other hand, SCN and MCN are commonly multilocular ant oligolocular, respectively. Nevertheless in cases of unilocular SCN and MCN, the preoperative differentitation with cystic NET without solid component might be impossible. In such cases, endoscopic ultrasound should be performed with fine needle aspiration of the content of the cyst [23].

The main reason for long-term postsurgical follow-up is the risk of recurrence [24]. The recommendation is to follow up these patients every 3–6 months for nonfunctional PanNETs and 6–12 months for functional PNETs (or 6–12 monthsaccording to the National Comprehensive Cancer Network (NCCN) every year for 3 years and every 1–2 years thereafter [24]. Singh et al. reported shorter disease-free survivals for PNET compared to other NETs and identification of tumor recurrence after 10 years of follow-up. All of this suggest that long-term follow-up is required for PNET. Cumulative incidence of recurrence was 26.5%, 39.6%, 57.0%, and 69.4% at 3, 5, 10, and 15 years post-resection, respectively [24,25].

Treatment of PNET depends on the stage of the disease at the moment of diagnosis with radical surgery being the most preferred modality in resectable cases [5]. For patients with localised disease, surgery is the treatment of choice, especially for G1 and G2 NETs. Since most of the cystic PNETs are non-functional and exhibit nonspecific symptoms, they are commonly discovered in later stages with distant metastasis [7]. Although primary tumor resection and liver-directed therapy procedures provide potential therapeutic benefits, additional systemic therapies may be required to reduce tumor burden for advanced PNETs. Current systemic treatment options represent the se of somatostatin analogs, chemotherapy, targeted therapy, and peptide receptor radionuclide therapy [26]. However, due to histopathologically proven G2 and low Ki-67 protein index, systemic therapy in our patient has not been considered. Nevertheless, even in advanced disease, patients show good survival rates. In our case, the patient had no metastasis, and total pancreatectomy with splenectomy was performed followed by successful postoperative recovery.

## 4. Conclusions

To our knowledge, this is the first case of synchronous occurrence of multiple cystic and solid NET diffusely distributed throughout pancreatic parenchyma. While preoperative diagnosis of solid PNET is usually straightforward, the diagnosis of cystic PNETs might pose a diagnostic dilemma. Peripheral rim enhancement in early arterial phase on CT and MRI is considered to be the most important imaging criteria for the diagnosis of cystic PNET. Timely diagnosis, precise diferentiation and multidisciplinary approach in the treatment of these lesions is necessary for improvement of patient outcomes.

## Data Availability

Not applicable.
